# Total Debranching Plus Antegrade Thoracic Endovascular Aortic Repair without Side Clamping in a Patient with Arch Aneurysm and Ascending Aorta Calcification

**DOI:** 10.3400/avd.cr.21-00035

**Published:** 2021-06-25

**Authors:** Ryutaro Isoda, Yuji Kanaoka, Tatsuya Watanabe, Atsuhisa Ishida, Masahiko Kuinose, Ichiro Morita

**Affiliations:** 1 Department of Surgery, Kawasaki Medical School General Medical Center, Okayama, Okayama, Japan; 2 Department of Cardiovascular Surgery, Kawasaki Medical School, Kurashiki, Okayama, Japan

**Keywords:** vascular calcification, clampless anastomosis, transaortic approach

## Abstract

A high-risk patient with aortic arch aneurysm, associated with severe calcification of the ascending aorta and iliac arteries, was treated with total debranching and antegrade thoracic endovascular aortic repair (TEVAR) via the ascending aorta. Proximal anastomosis for a triple-branched graft to the ascending aorta was performed without side clamping using the “real chimney technique.” After bypassing the supra-aortic branches, a TEVAR was performed in an antegrade fashion through the ascending aorta. This case suggests that the approaches mentioned above should be considered in patients with arch aneurysms and severe calcified degeneration.

## Introduction

Over the past decades, mortality and morbidity associated with the conventional open surgical repair (OSR) of aortic arch diseases in the standard at-risk population have decreased. However, OSR for aortic arch aneurysms remains challenging, particularly in patients with significant comorbidities, including advanced age and severe aortic calcification. Debranching plus thoracic endovascular aortic repair (TEVAR) has emerged as an OSR procedure for high-risk patients with aortic arch aneurysms.^1,2)^ When performing total debranching plus TEVAR, the supra-aortic branches are bypassed via the ascending aorta.^3)^ Side clamping of the ascending aorta is necessary for ascending aorta anastomosis; however, this also increases the rate of retrograde type A dissection and stroke.^4–6)^ Severe calcified degeneration of the ascending aorta and access route resulted in the emergence of the total debranching and subsequent standard retrograde approach for TEVAR. These challenges have been overcome by the “real chimney technique.”^7,8)^ We report the case of a patient with an arch aneurysm and severe calcified degeneration of the aorta and iliac arteries. Total debranching with clampless proximal anastomosis and TEVAR via the ascending aortic approach were successfully performed.

Written informed consent was obtained from the patient for the publication of this case report and any accompanying images.

## Ethical Approval

Ethical review board approval was waived because only non-identifiable data were used and because of the semi-emergency surgery caused by aneurysm condition.

## Case Report

**Figure 1:**
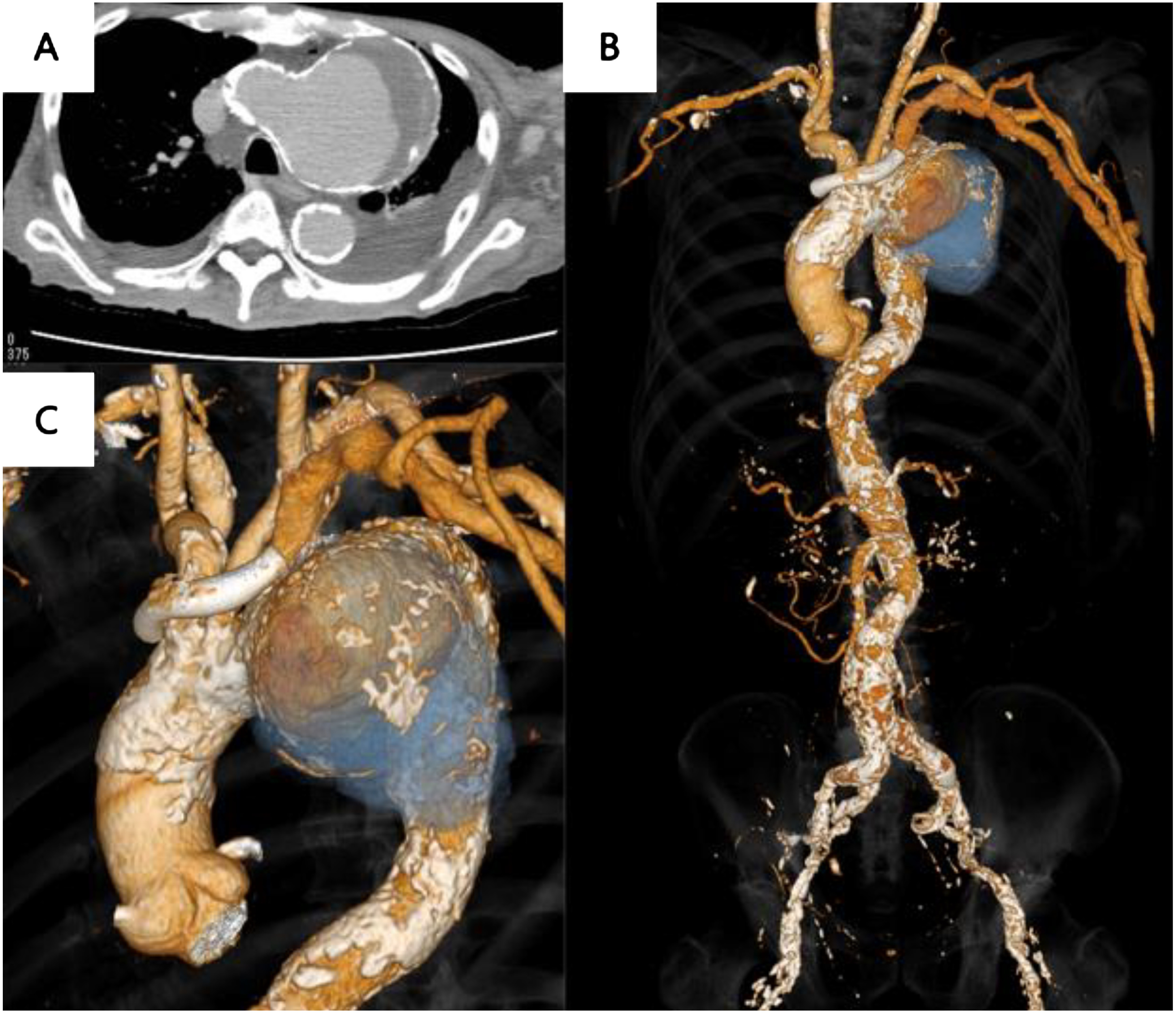
Fig. 1 (**A**) (**B**) Chest computed tomography (CT) scan reveals an aortic arch aneurysm with extremely severe aortoiliac calcification. (**C**) A bare-metal stent was inserted into the left brachiocephalic vein at another hospital because of arteriovenous fistula stenosis.

**Figure 2:**
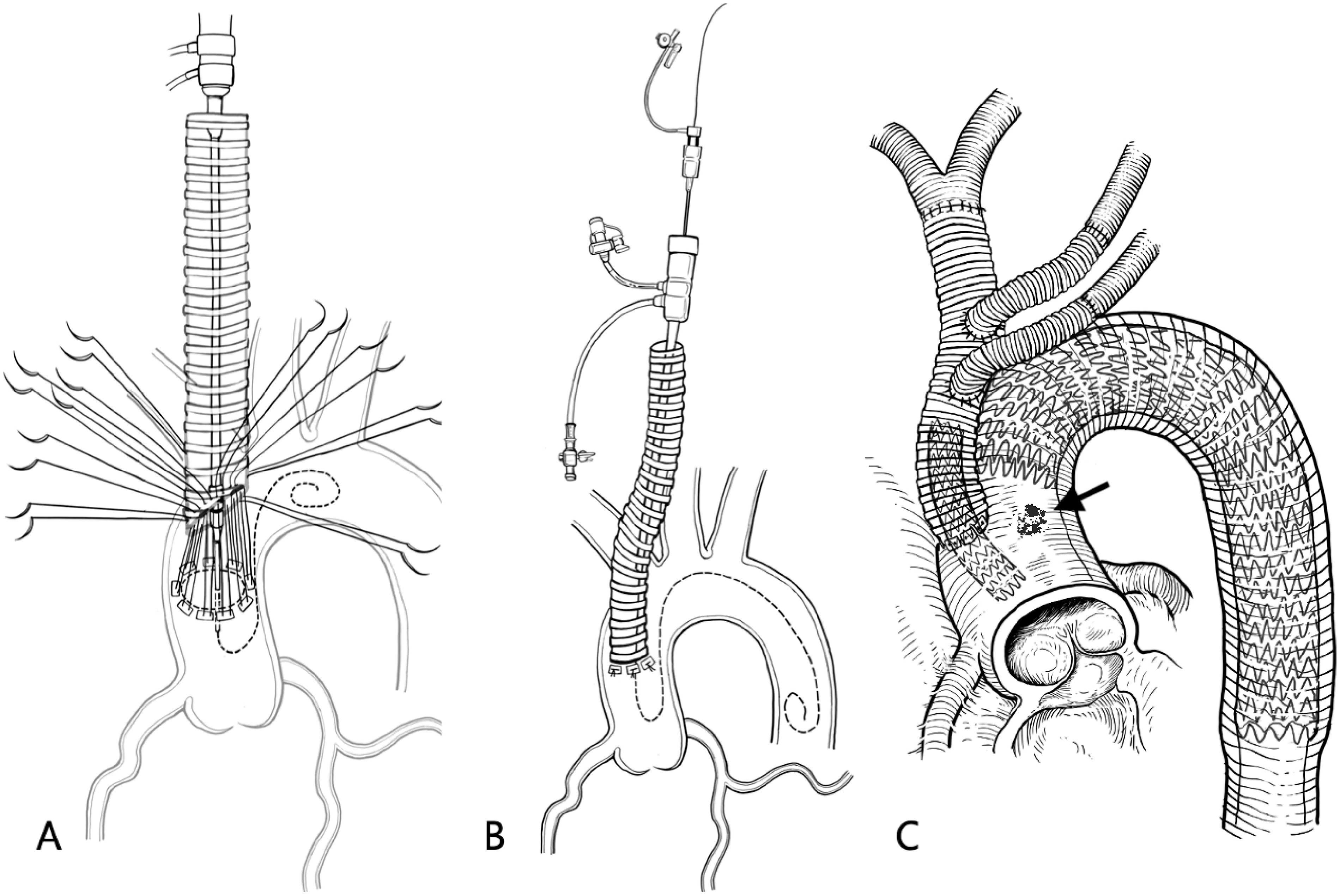
Fig. 2 (**A**) The prosthetic graft was fixed using mattress sutures with the ascending aorta. At that time, the center of the anastomosis site was punctured, and the guidewire was advanced to the descending aorta. (**B**) An 8-Fr sheath was inserted through the 10-Fr sheath fixed into the prosthetic graft. A VIABAHN VBX was deployed from the inside of the ascending aorta to the prosthetic graft. (**C**) Completion image of the total debranching using the real chimney technique plus thoracic endorascular aortic repair. The black arrow shows the stent graft insertion point.

**Figure 3:**
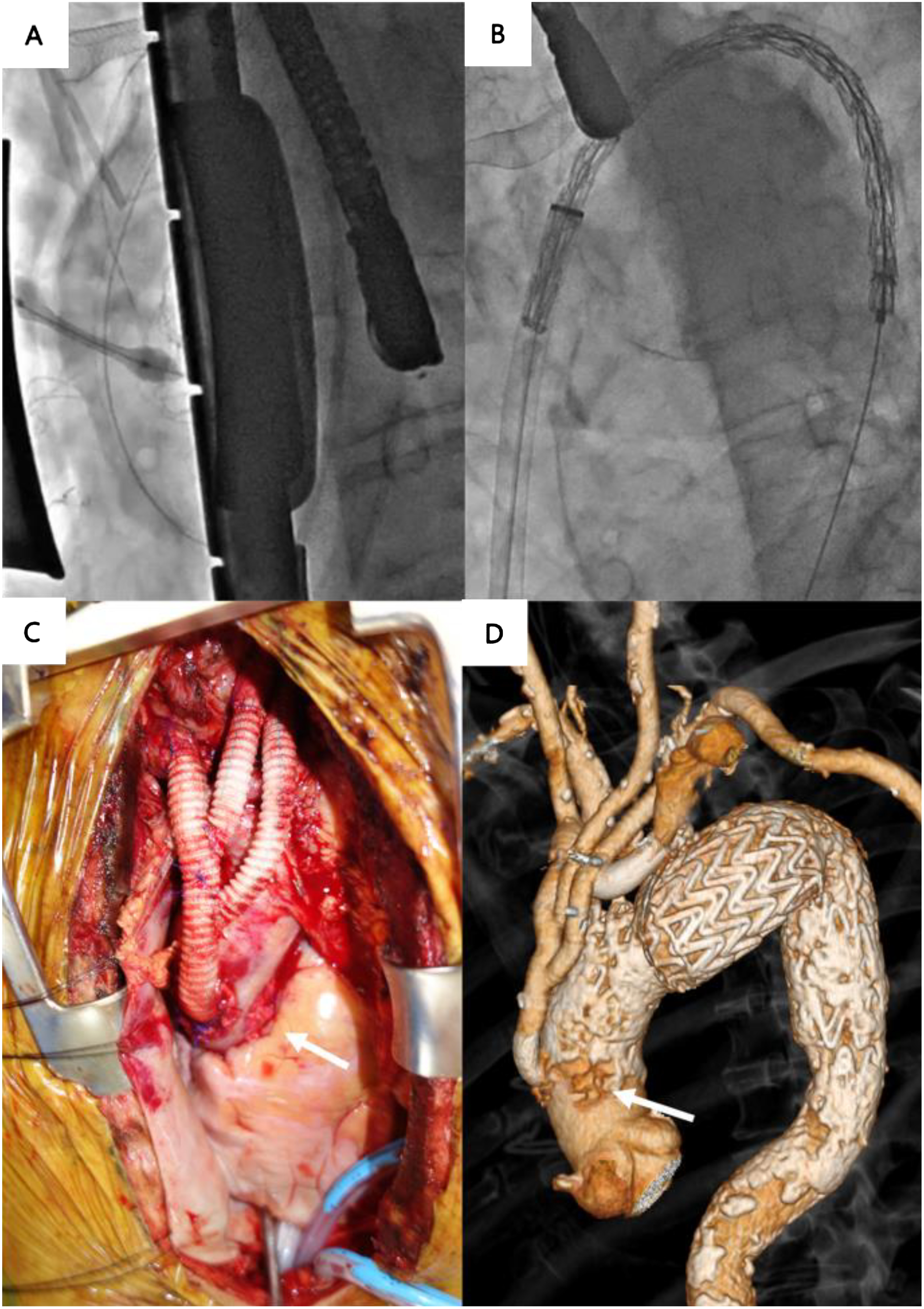
Fig. 3 (**A**) A VIABAHN VBX was placed into the proximal anastomosis. (**B**) A 24-Fr Dryseal sheath was placed, and conformable TAG was advanced in the ascending aorta. (**C**) Total debranching using the “real chimney technique.” The white arrow shows the device insertion point. (**D**) Postoperative computed tomography scans revealed successful exclusion of the aneurysm without an endoleak. The white arrow shows the device insertion point.

A 71-year-old man undergoing hemodialysis was referred to our hospital for an aortic arch aneurysm with hoarseness and chest discomfort. His medical history included hemodialysis for 12 years, chronic obstructive pulmonary disease, hypertension, and type 2 diabetes mellitus. An enhanced computed tomography (CT) scan revealed a 95-mm aortic arch aneurysm with a short proximal neck from the innominate artery, advanced calcified degeneration throughout the aorta, and severe aortoiliac obstructive calcification (**Fig. 1**). The patient had anatomical difficulties, such as severe calcification of the ascending aorta, the absence of the proximal neck, and severe stenotic iliofemoral access route with calcification. Although the patient was not a good candidate for intervention, surgical intervention was required because of his large symptomatic aneurysm. Therefore, total debranching plus TEVAR was performed according to the anatomical findings of the aorta. The anastomotic site in the calcification-free area of the ascending aorta was determined by manual examination after median sternotomy. Using a double-armed 4-0 polypropylene with a Teflon felt pledget, nine mattress sutures were placed in a circle at the ascending aorta. These nine mattress sutures were also used in a tube graft (9-mm-diameter woven polyester graft; Japan Lifeline Co., Ltd., Tokyo, Japan). Second, the center of the anastomosis site was punctured, and a 0.035-in radio focus guidewire (Terumo Corporation, Tokyo, Japan) was inserted into the descending aorta. The end of the guidewire was passed through the prosthetic graft (**Fig. 2A**). The prosthetic graft was fixed to the ascending aorta by tying the mattress sutures. Then, an 8-Fr sheath was inserted through the 10-Fr sheath fixed into the prosthetic graft (**Fig. 2B**). An 11×40 mm VIABAHN VBX balloon-expandable endoprosthesis (WL Gore & Associates, Flagstaff, AZ, USA) was telescoped inside the ascending aorta. The proximal 15 mm of the VIABAHN VBX was located inside the ascending aorta and deployed (**Fig. 3A**). Two 7-mm tube grafts were anastomosed to the 9-mm main graft in an end-to-side manner. Three distal anastomoses to the supra-aortic branches were completed in an end-to-end manner with the surgeon-made three-branched grafts (9–7-mm and 7-mm diameter woven polyester grafts) (**Fig. 3C**). After the bypass, two mattress sutures with Teflon felt pledget were made near the proximal anastomosis. A 24-Fr DrySeal sheath was directly inserted via the ascending aorta to the descending aorta using a Lunderquist guidewire (Cook Medical, Bloomington, IN, USA) (**Fig. 3B**). The initially conformable TAG (CTA G) (40×40×200 mm) (WL Gore & Associates, Flagstaff, AZ, USA) was deployed from the descending aorta of the seventh thoracic vertebral level of the arch. The second CTA G (40×40×200 mm) was deployed just distal to the proximal anastomosis of the ascending aorta. After removing the 24-Fr DrySeal sheath, two mattress sutures were tied. Angiography and postoperative CT scans showed successful bypass and exclusion of the aneurysm without any endoleak (**Fig. 3D**). No dissection or stroke occurred postoperatively. However, the general condition of the patient deteriorated because of pneumonia, and the patient died on postoperative day 31.

## Discussion

The clinical course of the patient provided two essential suggestions regarding the intervention. First, total debranching was performed without side clamping in a patient with an ascending aorta that should not or could not be side-clamped. The “real chimney technique” was performed for a vascular anastomosis on the ascending aorta without causing aortic dissection and stroke, despite the poor properties and advanced atherosclerosis of the ascending aorta. However, this method has a risk of aortic injury during ballooning of the VIABAHN VBX. As a countermeasure, the inflation pressure should be suppressed to about 6 atm to prevent aortic injury. Because the VIABAHN VBX was off-label use, it should be approved by the Institutional Review Board. However, the approval was waived because this case was a semi-emergency operation because of the aneurysm condition. Second, TEVAR was performed via the antegrade approach in a patient not indicated for the usual retrograde approach. Although the simplest antegrade approach was to insert the device through a prosthetic graft anastomosed to the ascending aorta,^8)^ accessing the aneurysm via the prosthetic graft was difficult in this case. Therefore, the “real chimney technique” was performed for the anastomosis. Furthermore, another access site in the ascending aorta was chosen, and a device was directly inserted after suturing with two mattresses with Teflon felt. Since the diameter of the device used for zone 0 landing was generally large, the antegrade approach of inserting the device from the ascending aorta was beneficial for total debranching and TEVAR.

## Conclusion

The approaches mentioned above should be considered in patients with arch aneurysms and severe calcified degeneration.
